# Ethynyl Radical Hydrogen Abstraction Energetics and Kinetics Utilizing High-Level Theory

**DOI:** 10.1021/acsearthspacechem.4c00040

**Published:** 2024-07-03

**Authors:** Laura N. Olive, Alexandra D. Heide, Justin M. Turney, Henry F. Schaefer

**Affiliations:** Center for Computational Quantum Chemistry, University of Georgia, Athens, Georgia 30602, United States

**Keywords:** ethynyl radical, hydrogen abstraction, kinetics, focal point analysis, theoretical study

## Abstract

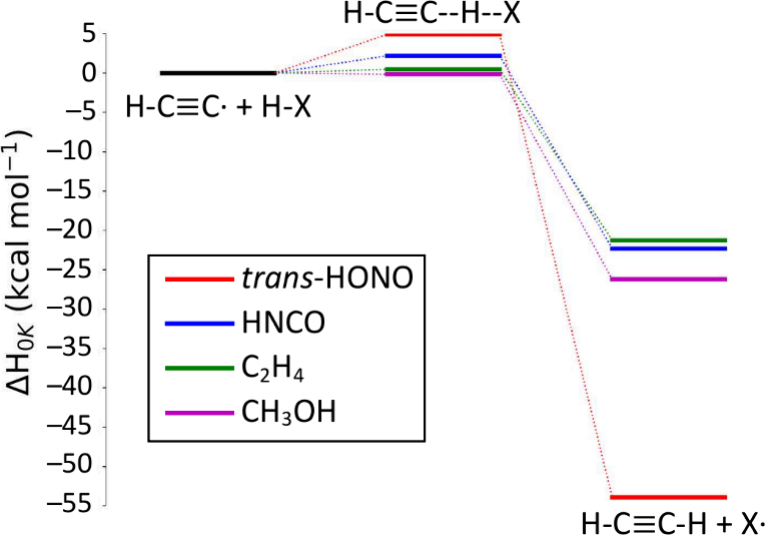

The ethynyl radical, C_2_H, is found in a variety of different environments ranging from interstellar space and planetary atmospheres to playing an important role in the combustion of various alkynes under fuel-rich conditions. Hydrogen-atom abstraction reactions are common for the ethynyl radical in these contrasting environments. In this study, the C_2_H + HX → C_2_H_2_ + X, where HX = HNCO, *trans*-HONO, *cis*-HONO, C_2_H_4_, and CH_3_OH, reactions have been investigated at rigorously high levels of theory, including CCSD(T)-F12a/cc-pVTZ-F12. For the stationary points thus located, much higher levels of theory have been used, with basis sets as large as aug-cc-pV5Z and methods up to CCSDT(Q), and core correlation was also included. These molecules were chosen because they can be found in either interstellar or combustion environments. Various additive energy corrections have been included to converge the relative enthalpies of the stationary points to subchemical accuracy (≤0.5 kcal mol^–1^). Barriers predicted here (2.19 kcal mol^–1^ for the HNCO reaction and 0.47 kcal mol^–1^ for C_2_H_4_) are significantly lower than previous predictions. Reliable kinetics were acquired over a wide range of temperatures (50–5000 K), which may be useful for future experimental studies of these reactions.

## 1 Introduction

The ethynyl radical, C_2_H (^2^Σ^+^), is known to be a key intermediate in a number of diverse environments. It has been observed in both combustion reactions^1,2^ and planetary atmospheres.^3,4^ Additionally, it is one of the most abundant polyatomic radicals in interstellar space.^5−8^ In combustion environments, the ethynyl radical is a common intermediate in fuel-rich hydrocarbon combustion processes of various alkynes that can take place at temperatures in excess of 1800 K.^9^ Furthermore, it plays an important role as a key precursor in the synthesis of polyynes, polycyclic aromatic hydrocarbons (PAHs), and soot particles.^1,10−13^ It is also a reactive species in Titan’s atmosphere where the temperature is altitude-dependent (70 K at the tropopause and 94 K at the surface). To better understand the importance of these reactions involving the ethynyl radical in these drastically different environments, there is a need to accurately model the reactions of C_2_H with a variety of reactants over an extremely broad range of temperatures.

Hydrogen-atom abstraction is among the most prevalent reactions for the ethynyl radical and is commonly the main reaction pathway or a feasible alternative.^14−16^ The sizable dissociation energy of acetylene’s sp-hybridized C–H bond thermodynamically drives these hydrogen abstraction reactions. Large rate constants have been calculated by kinetic studies for the reaction of C_2_H with different saturated hydrocarbons over a broad range of temperatures, implying moderately low barriers for abstraction.^2,9,17−19^ Theoretical results corroborate this claim with moderate to low reported barrier heights for several hydrogen atom donors.^14,20^ At this time, most experimental and theoretical studies involving ethynyl radical hydrogen abstractions have focused on hydrocarbon hydrogen atom donors. Over the years, numerous studies have been done on these types of reactions with a wide variety of small molecules or radicals.^2,11,14,15,21−30^ However, little work has been done on larger molecules.

Reactions involving nitrogen containing radicals and their kinetics in the gas phase are also of considerable interest due to the role these species play in the formation and removal of NO_*x*_ pollutants in combustion processes.^31^ NCO plays a key intermediate in combustion, however, very little is known of the kinetics of the reactions of NCO with hydrocarbons.^19,32^ No previous experimental studies on C_2_H + HNCO have been reported, but a theoretical study in 2003 by Chen and Ho^31^ addressed the reaction mechanisms for NCO and C_2_H_2_ at the CCSD(T)/6-31++G**//B3LYP/6-31++G** level of theory and they were able to find a direct pathway for the NCO + C_2_H_2_ → C_2_H + HNCO reaction.

Additionally, nitrous acid, HONO, has been detected in the interstellar medium,^33^ and the reaction involving CN has been explored theoretically^34^ with MP2 and CCSD(T); however, no study has been reported involving C_2_H. Furthermore, the kinetics of the pyrolysis and oxidation of methanol, CH_3_OH, which could be considered as an alternative, more environmentally friendly fuel or fuel additive to gasoline, have been of great interest in the last several years.^35^ Hydrogen abstraction reactions could happen at two different sites for CH_3_OH. The hydrogen could be abstracted off the hydroxyl, −OH, group (reaction R1), or the methyl, −CH_3_, group (reaction R2).
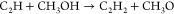
R1
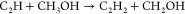
R2In 2011, a theoretical paper by Tri and Huê studied the C_2_H + CH_3_OH reaction mechanism with B3LYP and the 6-311++G(*d*,*p*) and 6-311++G(3*df*,2*p*) basis sets.^36^ They investigated 12 different reaction pathways and found that the formations of C_2_H_2_ + CH_3_O and C_2_H_2_ + CH_2_OH were the most favorable.

A recent study by Bowman et al. investigated numerous C_2_H hydrogen abstraction reactions with highly accurate *ab initio* methods.^15^ In the present study, a similar computational approach will be utilized to investigate the C_2_H + HX → C_2_H_2_ + X reactions, where HX = HNCO, *trans*-HONO, *cis*-HONO, C_2_H_4_, and CH_3_OH. Our study will provide high-level *ab initio* characterization of hydrogen abstraction reactions of the ethynyl radical with various medium sized molecules including HNCO, *trans*-HONO, *cis*-HONO, C_2_H_4_, and CH_3_OH. A composite approach will be implemented to converge the energies within subchemical accuracy (≤0.5 kcal mol^–1^). These highly accurate energetics will be used to compute reliable rate constants with canonical transition state theory that can be used in future kinetic studies.

## 2 Theoretical Methods

Full geometry optimizations and corresponding harmonic vibrational frequency computations were performed on each of the stationary points for the C_2_H + HX, where HX = HNCO, *trans*-HONO, C_2_H_4_, and CH_3_OH (reaction R1), reactive surfaces using the explicitly correlated CCSD(T)-F12a method^37^ in conjuction with the cc-pVTZ-F12 basis set^38,39^ as implemented in Molpro 2010.^40^ A restricted open-shell Hartree–Fock (ROHF) reference was used for all open-shell computations to avoid issues with spin contamination that are sometimes prevalent with the ethynyl radical. For the C_2_H + HX, where HX = *cis*-HONO and CH_3_OH (reaction R2), reactive surfaces, full geometry optimizations and corresponding harmonic vibrational frequency computations were performed on each of the stationary points at the MP2^41^/aug-cc-pVTZ^42^ level of theory as implemented in Psi4.^43^ Single point energy computations were performed on the MP2/aug-cc-pVTZ geometries with CCSD(T)-F12a/cc-pVTZ-F12 in Molpro 2010.

The electronic energies of the CCSD(T)-F12a/cc-pVTZ-F12 stationary points were computed according to the focal point analysis (FPA) of Allen and co-workers.^44,45^ For the present study, methods that describe electron correlation up to CCSDT(Q)^46^ and basis sets as large as aug-cc-pV5Z^47^ were used. Single point energy computations were performed on the CCSD(T)-F12a/cc-pVTZ-F12 optimized geometries with CCSD(T)^48,49^/aug-cc-pV*X*Z (where *X* = D, T, Q, 5)^42^ in Molpro 2010, and CCSDT^50^/aug-cc-pVDZ and CCSDT(Q)/aug-cc-pVDZ as implemented in MRCC 2018.^51^ As shown in Table 1, there is excellent convergence to the complete basis set (CBS) limit. The CCSD(T)/CBS energies were obtained through extrapolation of the Hartree–Fock (HF) and correlation energies. The three-parameter exponential function by Feller is used to extrapolate to the HF/CBS limit.^52^
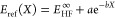
1The two-parameter cubic function of Helgaker et al.^53^ is used to extrapolate the correlation energies (*E*_corr_) to the CBS limit.
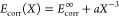
2The focal point energies were obtained with the following formula:
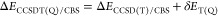
3Additional corrections were made to account for approximations made during the focal point computations. To account for the core-correlation neglected under the frozen-core approximation, the CCSD(T)/aug-cc-pCVQZ energy with all electrons correlated was computed and the difference between the energies with and without the core–electrons was determined (δ_CORE_). A scalar relativistic correction (δ_REL_) was obtained at the X2C–CCSD(T)/aug-cc-pCVTZ-X2C level of theory.^54^ The clamped-nuclei approximation was treated with the diagonal Born–Oppenheimer correction (δ_DBOC_)^55,56^ performed at the ROHF/aug-cc-pVTZ level of theory. Spin–orbit coupling constants (δ_SO_) were included for the NCO and CH_3_O products in order to account for the splitting of the electronic ground state.^57,58^ Lastly, zero-point vibrational energies (δ_ZPVE_) were obtained from the CCSD(T)-F12a/cc-pVTZ-F12 harmonic vibrational frequencies. All of these corrections were added together to obtain the relative enthalpies at 0 K using the following equation:
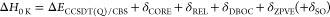
4Using canonical transition state theory,^59,60^ the rate constants were computed over a wide range of temperatures
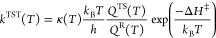
5where *Q*^TS^(*T*) and *Q*^R^(*T*) are the partition functions of the transition state and reactants, respectively, and Δ*H*^⧧^ is the reaction barrier height from eq 4. The transmission coefficient, κ(*T*), was determined with an asymmetric Eckart potential barrier using the relative enthalpies of the prereactive complex, transition state, and products for each reaction, and the imaginary harmonic vibrational frequency corresponding to the reaction mode of the transition state.^61^

**Table 1:** Representative Incremental Focal Point Table for the Products of the C_2_H + HNCO → C_2_H_2_ + NCO Reaction Relative to the Reactants (kcal mol^–1^)^a^

basis set	HF	+δMP2	+δCCSD	+δ(T)	+δT	+δ(Q)	net
aug-cc-pVDZ	–23.32	+4.64	–4.01	+0.30	–0.10	–0.05	[−22.54]
aug-cc-pVTZ	–23.54	+4.52	–4.20	+0.27	[−0.10]	[−0.05]	[−23.09]
aug-cc-pVQZ	–23.34	+4.76	–4.11	+0.26	[−0.10]	[−0.05]	[−22.58]
aug-cc-pV5Z	–23.33	+4.80	–4.08	+0.25	[−0.10]	[−0.05]	[−22.51]
CBS limit	[−23.34]	[+4.83]	[−4.05]	[+0.25]	[−0.10]	[−0.05]	[−22.46]

a Additional focal point tables can be found in the Supporting Information. δ denotes the change in the relative energy with respect to the previous level of theory. The numbers in [ ] are obtained by the extrapolation schemes found in the Theoretical Methods section.

Bowman and co-workers demonstrated that an Eckart tunneling model provides accurate kinetics in agreement with experimental results for hydrogen abstraction reactions involving the ethynyl radical;^15^ therefore, Eckart tunneling was used here. Additionally, other studies have achieved past success in accurately describing the tunneling of hydrogen transfer reactions at moderate to high temperatures using Eckart tunneling.^62−64^ In this work, pressure dependence will not be taken into account.

## 3 Results and Discussion

### 3.1 Energies and Geometries

Table 2 shows the reaction enthalpies at 0 K for the C_2_H hydrogen-abstractions involving HNCO, *trans*-HONO, C_2_H_4_, and CH_3_OH (reaction R1). The last column of Table 2 demonstrates excellent agreement between our computed reaction enthalpies and the reaction enthalpies reported in the Active Thermochemical Tables (ATcT) (Version 1.122r) of Ruscic and co-workers.^65,66^ The mean absolute error (MAE) between the two is 0.19 kcal mol^–1^. The root-mean-square error (RMSE) is 0.25 kcal mol^–1^. The largest deviation is found for the CH_3_OH + C_2_H → CH_3_O + C_2_H_2_ reaction with a difference of 0.48 kcal mol^–1^.

**Table 2:** Enthalpies at 0 K (Δ*H*_0 K_ in kcal mol^–1^ for Products Relative to Reactants (C_2_H + HX → C_2_H_2_ + X)^a^

donor	CBS^b^	δ_T(Q)_	δ_CORE_	δ_REL_	δ_DBOC_	δ_ZPVE_	δ_SO_	total	ATcT^c^	Δ|*E*|^d^
HNCO	–22.31	–0.15	0.08	–0.16	0.00	0.63	–0.14	–22.05	–22.02	0.03
*trans*-HONO	–53.93	0.15	–0.47	0.02	0.00	0.53		–53.69	–53.82	0.13
C_2_H_4_	–21.25	0.13	–0.17	–0.02	–0.04	–1.24		–22.58	–22.68	0.10
CH_3_OH^e^	–26.22	0.21	–0.20	–0.14	0.47^f^	–1.49	–0.41	–28.25	–27.77	0.48

a δ denotes various corrections. See the Theoretical Methods section for details.

b CBS denotes the CCSD(T)/CBS relative energy.

c Enthalpies obtained from the ATcT.^65,66^

d Absolute value of the difference between total and ATcT.

e CH_3_OH + C_2_H → CH_3_O + C_2_H_2_.

f DBOC correction not included in the final total.

**Figure 1:**
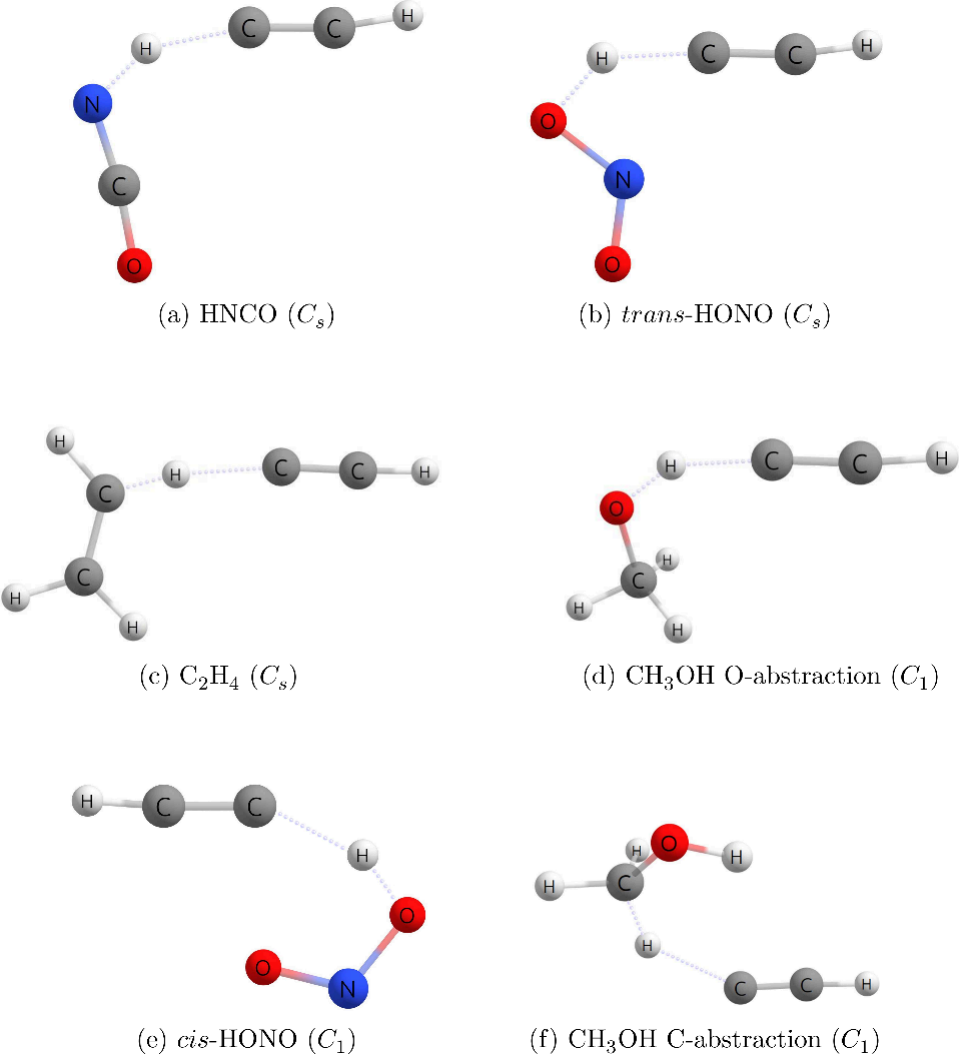
Qualitative geometries of C_2_H + HX transition states.

Figure 1 depicts the geometries of the hydrogen abstraction transition states found for each reaction studied in this research. Table 3 shows the reaction enthalpies of the CCSD(T)-F12a/cc-pVTZ-F12 transition states relative to their respective reactants. According to the results in Table 2, the energetics of the products are expected to be accurate within 0.48 kcal mol^–1^; however, the relative energetics of the transition states are highly dependent on the employed level of theory. Because of this, we expect the barrier heights to be reliable well within chemical accuracy (1 kcal mol^–1^). Of the corrections listed in Table 3, δ_ZPVE_ is the largest suggesting that obtaining accurate barrier heights not only requires accurate electronic energies, but also reliable vibrational frequencies. Additionally, δ_REL_ is small, but not negligible for these reactions which involve first and second row atoms. The diagonal Born–Oppenheimer corrections for the reaction enthalpies and transition state barriers were consistently small (≤0.2 kcal mol^–1^) for almost all of the reactions. However, for the CH_3_OH + C_2_H → CH_3_O + C_2_H_2_ reaction where δ_DBOC_ is computed as 0.47 kcal mol^–1^. Bartlett and co-workers proposed that δ_DBOC_ can be utilized as a diagnostic for the presence of a nearby conical intersection.^67^ Typically for most well-behaved systems without a nearby conical intersection, the δ_DBOC_ is small, but this is not true in the proximity of a conical intersection. The *E*_DBOC_ becomes nonintegrable over domains that include a conical intersection because the second-derivative of the *T̂*_*n*_ operator blows up, as shown by Meek and Levine.^68^ It is recommended that DBOC not be included when employing mixed quantum-classical methods and approximate quantum dynamical methods. Therefore, the δ_DBOC_ was not included in determining the final reaction enthalpy and transition state barrier of the CH_3_OH + C_2_H → CH_3_O + C_2_H_2_ reaction.

**Table 3:** Enthalpies at 0 K (Δ*H*_0 K_ in kcal mol^–1^ for Transition States Relative to Reactants (C_2_H + HX → C_2_H_2_ + X)^a^

donor	CBS^b^	δ_T(Q)_	δ_CORE_	δ_REL_	δ_DBOC_	δ_ZPVE_	total
HNCO	4.06	–0.19	0.14	–0.14	0.23	–1.90	2.19
*trans*-HONO	6.38	–0.96	0.03	0.23	–0.01	–0.76	4.91
C_2_H_4_	1.74	–0.11	–0.01	–0.01	0.02	–1.17	0.47
CH_3_OH^c^	1.49	–0.28	0.01	–0.21	0.11^d^	–1.28	–0.27

a δ denotes various corrections. See the Theoretical Methods section for details.

b CBS denotes the CCSD(T)/CBS relative energy.

c CH_3_OH + C_2_H → CH_3_O + C_2_H_2_.

d DBOC correction not included in the final total.

As shown in Table 3, the C_2_H + CH_3_OH reaction has a very slightly submerged transition state barrier of −0.27 kcal mol^–1^, suggesting that this reaction will be rapid even at low temperatures. The C_2_H + HNCO and C_2_H + *trans*-HONO reactions have low barriers less than 5 kcal mol^–1^, meaning these reactions will likely proceed more slowly than the barrierless reaction.

**Figure 2:**
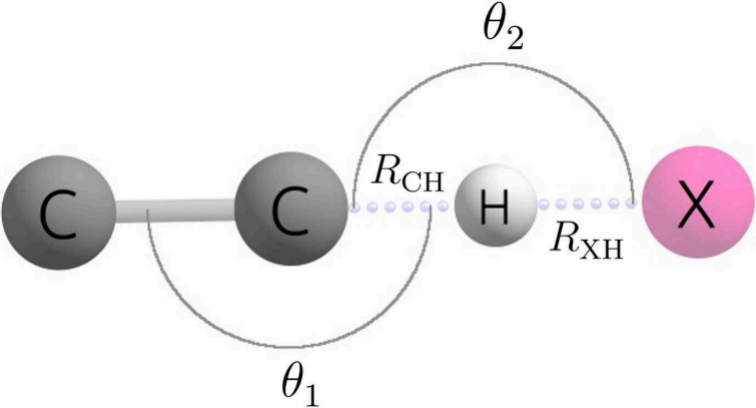
General representation of the transition state geometrical features.

Table 4 compares the reaction enthalpies (Δ_r_*H*), the barrier heights (Δ*H*^⧧^), important transition state features (shown in Figure 2), and the imaginary mode frequency (ω^⧧^) of the transition state of the C_2_H + HX, where HX = HNCO, *trans*-HONO, C_2_H_4_, and CH_3_OH (reaction R1), reactions. To better determine if these hydrogen abstraction reactions follow the Evans–Polanyi principle, the reactions have been listed in order of decreasing exothermicity (Δ_r_*H*). The Evans–Polanyi principle observes that the activation energy between two similar reactions is inversely proportional to the reaction exothermicity.^69,70^ The reactions studied here, in general, appear to follow the Evans–Polanyi principle; however, the C_2_H + *trans*-HONO reaction seems to be an exception. One might expect the abstraction of the hydrogen from *trans*-HONO to have a submerged barrier of around −2 kcal mol^–1^. Instead, the barrier height for *trans*-HONO is 4.91 kcal mol^–1^. The interaction between the nitrogen of *trans*-HONO and the terminal carbon of C_2_H could potentially be causing the unexpected Δ*H*^⧧^ increase.

**Table 4:** Reaction Enthalpies (Δ^r^*H*), Reaction Barrier Heights (Δ*H*^⧧^), and Transition State Imaginary Frequencies (ω^⧧^) for C_2_H + HX Hydrogen Abstractions at the CCSDT(Q)/CBS//CCSD(T)-F12a/cc-pVTZ-F12 Level of Theory

donor	Δ_r_*H*	Δ*H*^⧧^	*R*_CH_	Δ*R*_XH_ (%)^a^	θ_1_	θ_2_	ω^⧧^
*trans*-HONO	–53.7	4.9	1.480	17.7	176.2	133.2	1703*i*
CH_3_OH^b^	–27.8	–0.3	1.540	6.8	174.4	134.4	794*i*
C_2_H_4_	–22.6	0.5	1.653	4.7	173.4	168.7	267*i*
HNCO	–22.1	2.2	1.500	9.1	168.2	145.9	947*i*

a Δ*R*_XH_ = (*R*_XH,TS_ – *R*_XH,eq_)/*R*_XH,eq_.

b CH_3_OH + C_2_H → CH_3_O + C_2_H_2_.

To better understand the relationship between the reaction rates and how closely the transition states match the isolated reactants, Hammond’s postulate is assessed. The Hammond idea states that the geometry of the transition state resembles either the reactants or products depending on the exothermicity of the reaction, and this was also taken into consideration in this study. In order to determine how closely the transition state resembles the isolated reactants, the distance between the terminal carbon of the ethynyl radical and the hydrogen that is being abstracted in the transition state (*R*_CH_), was considered. The percent change between the XH bond length in the donor bond and the transition state (Δ*R*_XH_) was also taken into account. The transition state will more closely resemble the reactants than the products if the Δ*R*_XH_ value is less than 50%. The reactions in this study do follow Hammond’s postulate. For example, the Δ*R*_XH_ value of the C_2_H + HNCO reaction is 9.1%, and the energy difference of the reactants and transition state is 2.2 kcal mol^–1^ while the energy difference of the transition state and products is 24.3 kcal mol^–1^. Energetically, the transition state lies closer to the reactants than the products, which corresponds to the calculated Δ*R*_XH_ value that is less than 50%.

#### 3.1.1 C_2_H + HNCO

A previous study by Chen and Ho investigated the C_2_H + HNCO → C_2_H_2_ + NCO reaction at the CCSD(T)/6-311++G**//B3LYP/6-311++G** level of theory.^31^ They found that the reaction proceeded through a transition state barrier of 6.23 kcal mol^–1^ and ended with the products at −21.79 kcal mol^–1^ relative to the reactants.

**Figure 3:**
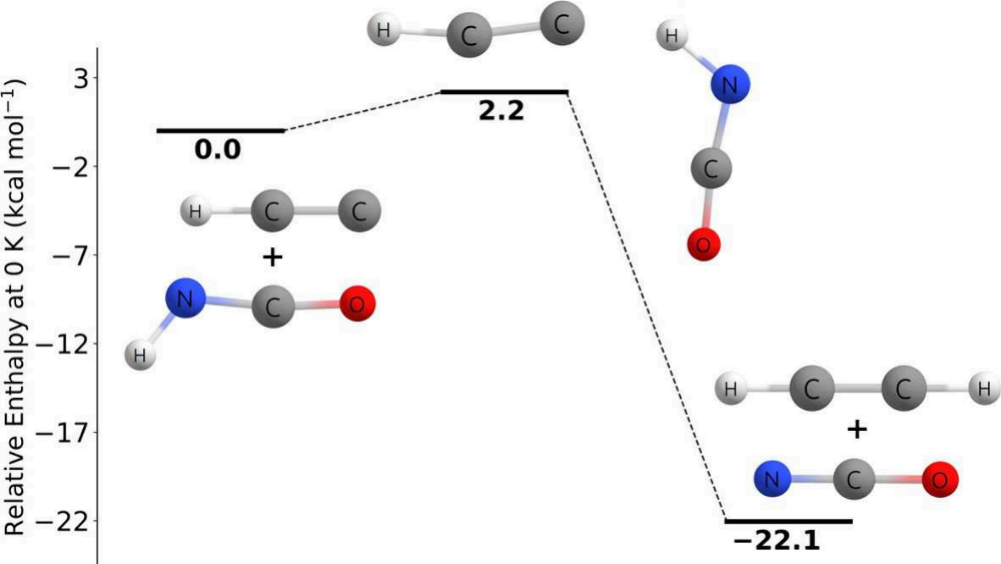
Potential energy surface of the C_2_H + HNCO → C_2_H_2_ + NCO reaction at 0 K at the CCSDT(Q)/CBS//CCSD(T)-F12a/cc-pVTZ-F12 level of theory.

As seen in Figure 3, the reaction pathway found in this study is qualitatively similar to that of Chen and Ho. As laid out in Table 5, our transition state barrier of 2.19 kcal mol^–1^ is 4.04 kcal mol^–1^ lower than that of Chen and Ho. However, the ending products of −22.05 kcal mol^–1^ relative to reactants show less than a 0.3 kcal mol^–1^ difference. The bond lengths of the transition state geometry are also similar to that of Chen and Ho with the largest difference being 0.14 Å for the H–C bond between HNCO and C_2_H. However, there is an 11.5° difference for the CNH angle in HNCO. Chen and Ho did not report rate constants for this reaction.

**Table 5:** Comparison of the C_2_H + HNCO → C_2_H_2_ + NCO Abstraction at Different Levels of Theory^a^

	Δ*H*^⧧^	Δ*H*_r_	*R*_CH_	*R*_NH_	θ_CHN_
Chen and Ho^b^	6.23	–21.79	1.644	1.071	129.29
this work^c^	2.19	–22.05	1.500	1.096	117.77

a Enthalpies are given in kcal mol^–1^; bond distances are given in Å; and angles are given in degrees.

b CCSD(T)/6-31++G**//B3LYP/6-31++G**.

c CCSDT(Q)/CBS//CCSD(T)-F12a/cc-pVTZ-F12.

#### 3.1.2 C_2_H + HONO

Both the *trans* and *cis* isomers of HONO were considered for this study; however, only the reaction pathway involving the *trans* isomer was characterized at the highest level of theory implemented in this study. The study that investigated the CN + HONO reaction found that the *trans* isomer is 0.45 kcal mol^–1^ lower in energy than the *cis* isomer with an isomerization barrier of 9.46 kcal mol^–1^ at the CCSD(T)/aug-cc-pVTZ//UMP2/6-311++G(*d*,*p*) level of theory.^34^

**Figure 4:**
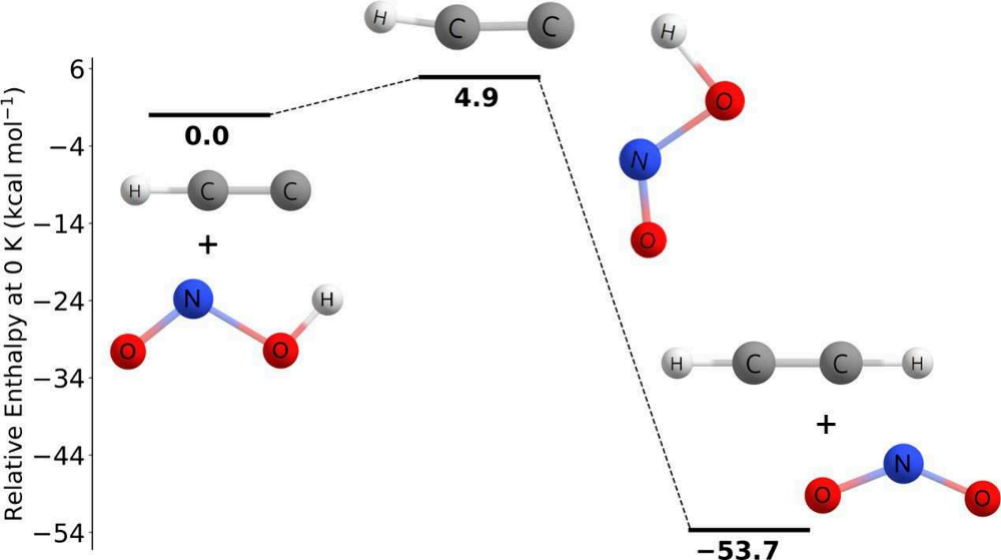
Potential energy surface of the C_2_H + *trans*-HONO → C_2_H_2_ + NO_2_ reaction at 0 K at the CCSDT(Q)/CBS//CCSD(T)-F12a/cc-pVTZ-F12 level of theory.

**Figure 5:**
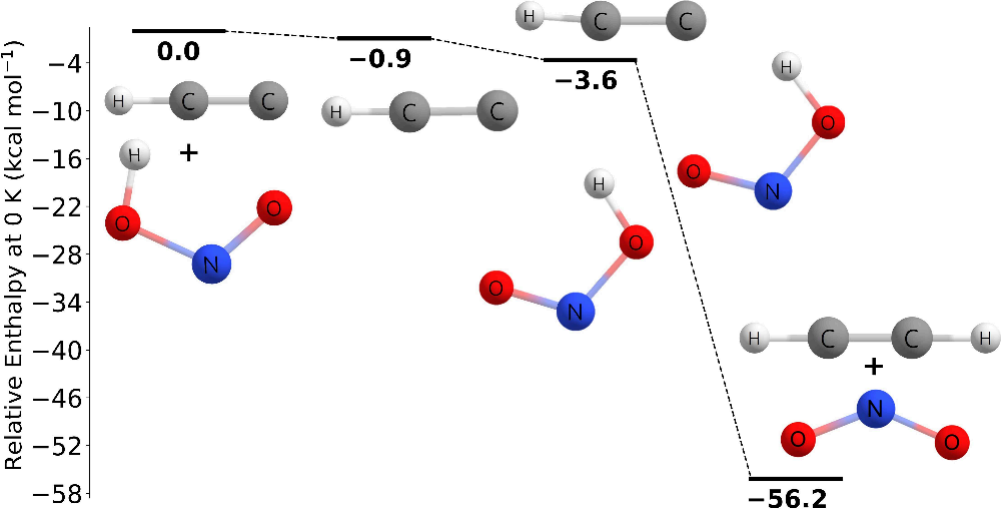
Potential energy surface of the C_2_H + *cis*-HONO → C_2_H_2_ + NO_2_ reaction at 0 K at the CCSD(T)-F12a/cc-pVTZ-F12//MP2/aug-cc-pVTZ level of theory.

There have been no previously reported theoretical studies or experimental measurements involving the C_2_H + HONO → C_2_H_2_ + NO_2_ reaction. As shown in Figure 4, the reaction pathway involving the *trans* isomer found in this work at the CCSDT(Q)/CBS//CCSD(T)-F12a/cc-pVTZ-F12 level of theory proceeds through a transition state barrier of 4.91 kcal mol^–1^. Due to the stabilities of the ethylene and nitrogen dioxide, the energy of the products is −53.69 kcal mol^–1^ relative to the reactants. In Figure 5, the reaction pathway involving the *cis* isomer found in this work at the CCSD(T)-F12a/cc-pVTZ-F12//MP2/aug-cc-pVTZ level of theory proceeds through a prereactive complex at −0.91 kcal mol^–1^ followed by a submerged transition state barrier of −3.62 kcal mol^–1^ and ends with the products at −56.17 kcal mol^–1^ relative to the reactants.

#### 3.1.3 C_2_H + CH_3_OH

There have been no previously reported experimental measurements involving the C_2_H + CH_3_OH → C_2_H_2_ + CH_3_O/CH_2_OH reactions. One previous study by Tri and Huê investigated the C_2_H + CH_3_OH reaction mechanism theoretically and determined the potential energy surfaces of 12 different reaction pathways.^36^ They determined that the pathways that formed C_2_H_2_ + CH_3_O and C_2_H_2_ + CH_2_OH were the most favorable with submerged barrier heights of −4.01 and −0.14 kcal mol^–1^, and ended with the products at −32.32 and −38.45 kcal mol^–1^, respectively, at the B3LYP/6-311++G(3*df*,2*p*)//B3LYP/6-311++G(*d*,*p*) level of theory. For the C_2_H + CH_3_OH → C_2_H_2_ + CH_3_O reaction, they were able to locate a prereactive complex at −4.06 kcal mol^–1^, but they did not report a prereactive complex for the C_2_H + CH_3_OH → C_2_H_2_ + CH_2_OH reaction.

**Figure 6:**
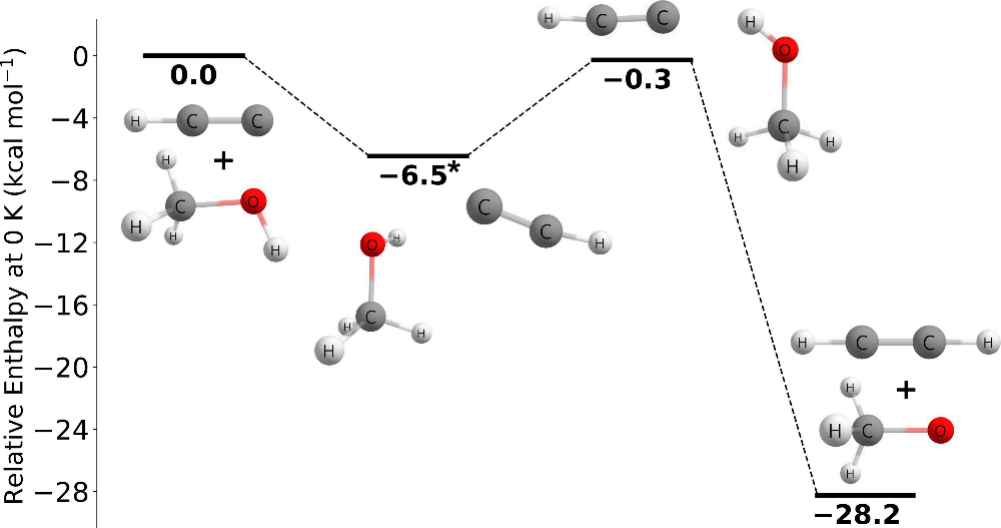
Potential energy surface of the C_2_H + CH_3_OH → C_2_H_2_ + CH_3_O reaction at 0 K at the CCSDT(Q)/CBS//CCSD(T)-F12a/cc-pVTZ-F12 level of theory. The asterisk (∗) denotes the energy is at the CCSD(T)-F12a/cc-pVTZ-F12 level of theory with no additional corrections.

As shown in Figure 6, the C_2_H + CH_3_OH → C_2_H_2_ + CH_3_O reaction proceeds through a prereactive complex at −6.45 kcal mol^–1^ and then through a very slightly submerged transition barrier of −0.27 kcal mol^–1^, and ends in the products at −28.25 kcal mol^–1^ relative to the reactants, which is qualitatively in agreement to that of Tri and Huê. The prereactive complex energy was determined at the CCSD(T)-F12a/cc-pVTZ-F12 level of theory with no additional corrections.

**Figure 7:**
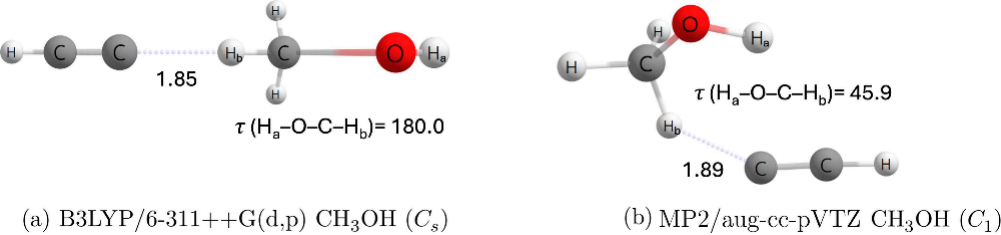
Qualitative geometries of C_2_H + CH_3_OH methyl abstraction transition state. Bond distances are given in Å, and angles are given in degrees.

Additionally, the C_2_H + CH_3_OH → C_2_H_2_ + CH_2_OH reaction was also investigated for this study; however, no direct transition state was found for CH_2_OH production at our highest-level of theory. The reported transition state geometry of Tri and Huê appears to be in *C*_*s*_ symmetry (Figure 7a) with a H_*a*_–O–C-H_*b*_ dihedral angle of 180.0°. Calculations at the MP2/aug-cc-pVTZ level of theory determined a transition state with a H_*a*_–O–C-H_*b*_ dihedral angle of 45.9° (Figure 7b). This floppy dihedral angle made this transition state difficult to optimize and locate.

**Figure 8:**
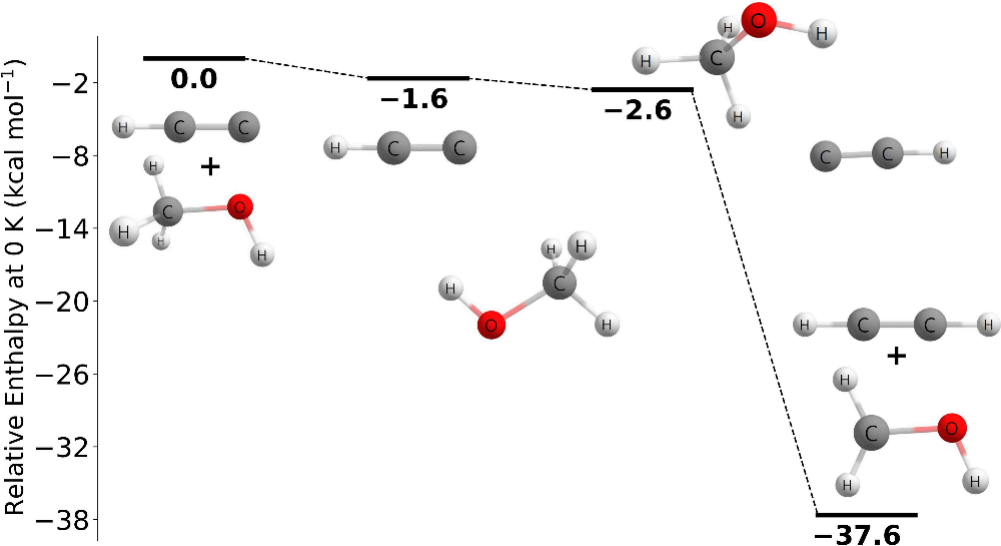
Potential energy surface of the C_2_H + CH_3_OH → C_2_H_2_ + CH_2_OH reaction at 0 K at the CCSD(T)-F12a/cc-pVTZ-F12//MP2/aug-cc-pVTZ level of theory.

As shown in Figure 8, the C_2_H + CH_3_OH → C_2_H_2_ + CH_2_OH reaction pathway characterized at the CCSD(T)-F12a/cc-pVTZ-F12//MP2/aug-cc-pVTZ level of theory proceeds through a prereactive complex at −1.64 kcal mol^–1^ followed by a submerged transition state barrier of −2.58 kcal mol^–1^ and ends with the products at −37.63 kcal mol^–1^ relative to the reactants. MP2/aug-cc-pVTZ gives an imaginary mode of 32*i* for the prereactive complex, but we believe this mode is an artifact of the level of theory and will likely disappear at a more rigorous level of theory. In terms of chemical reactivity, it appears this reaction pathway where the hydrogen is abstracted from the methyl group will likely prevail because it has a lower barrier height compared to the hydrogen being abstracted from the hydroxyl group. However, Tri and Huê report that the reaction pathway where the hydrogen is abstracted from the hydroxyl group has a lower transition state barrier; therefore, it would be beneficial to further study this reaction pathway in a future study.

#### 3.1.4 C_2_H + C_2_H_4_

A previous study by Temelso and co-workers studied the C_2_H + C_2_H_4_ → C_2_H_2_ + C_2_H_3_ reaction with the B3LYP, BHLYP, MP2, and CCSD(T) methods in conjunction with the cc-pV*X*Z (where *X* = D, T, Q) basis sets.^71^ However, CCSD(T)/cc-pVDZ was the highest level of theory reported for the barrier height. They found that the reaction proceeds through a transition barrier of 1.7 kcal mol^–1^, and ends in the products at −19.5 kcal mol^–1^ at the CCSD(T)/cc-pVDZ level of theory using an ROHF reference. They also calculated the Δ*H*(0 K) at the CCSD(T)/cc-pVTZ level of theory and determined it to be −22.6 kcal mol^–1^, which is in excellent agreement with our results.

**Figure 9:**
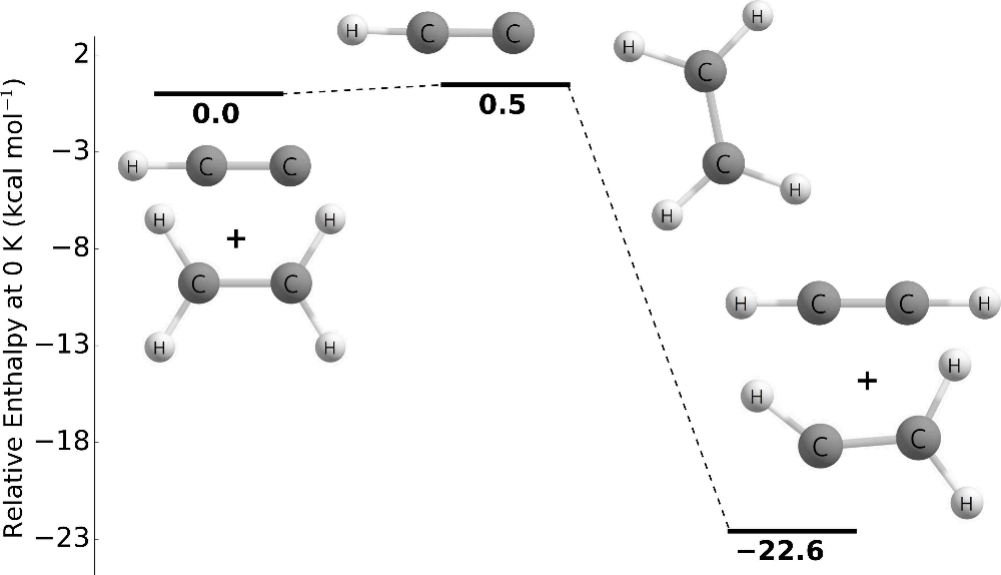
Potential energy surface of the C_2_H + C_2_H_4_ → C_2_H_2_ + C_2_H_3_ reaction at 0 K at the CCSDT(Q)/CBS//CCSD(T)-F12a/cc-pVTZ-F12 level of theory.

As shown in Figure 9, the reaction pathway characterized in this study is qualitatively similar to that of Temelso and co-workers. Our transition state barrier of 0.47 kcal mol^–1^ is 1.23 kcal mol^–1^ lower than that of Temelso and co-workers, but their ending product energy of −22.6 kcal mol^–1^ relative to reactants at the CCSD(T)/cc-pVTZ level of theory is in excellent agreement with our results. The transition state geometries are quite similar as shown in Table 6, with the largest difference being the CHC angle. Temelso and co-workers report a linear angle while we predict a 168.7 degree angle. Rate constants were not reported for this reaction by Temelso and co-workers.

**Table 6:** Comparison of the C_2_H + C_2_H_4_ → C_2_H_2_ + C_2_H_3_ Abstraction at Different Levels of Theory^a^

	Δ*H*^⧧^	Δ*H*_r_	*R*_CH_	*R*_CH(2)_	θ_CHC_
this work^b^	0.5	–22.6	1.653	1.133	168.7
Temelso^c^	1.7	–22.6^d^	1.610	1.155	180.0
Dash and Rajakumar^e^	0.3	–21.9	1.653	1.136	172.0
Dash and Rajakumar^f^	2.3				
Dash and Rajakumar^g^	–1.4	–23.0			

a Enthalpies are given in kcal mol^–1^; bond distances are given in Å; and angles are given in degrees. *R*_CH(2)_ is the C–H bond distance to H that is being abstracted from C_2_H_4_.

b CCSDT(Q)/CBS//CCSD(T)-F12a/cc-pVTZ-F12.

c CCSD(T)/cc-pVDZ with an ROHF reference.

d CCSD(T)/cc-pVTZ with an ROHF reference.

e M06-2X/6-31+G(*d*,*p*).

f CCSD(T)/cc-pVTZ//M06-2X/6-31+G(*d*,*p*).

g G3(MP2)//M06-2X/6-31+G(*d*,*p*).

Additionally, Dash and Rajakumar studied the C_2_H + C_2_H_4_ → C_2_H_2_ + C_2_H_3_ reaction and computed the CCSD(T)/cc-pVTZ and G3(MP2) electronic energies at each stationary point. However, they employed the M06-2X/6-31+G(*d*,*p*) level of theory to optimize the geometries and determine the harmonic vibrational frequencies.^20^ The transition state barrier is quite different at each level of theory as seen in Table 6. The M06-2X/6-31+G(*d*,*p*) pathway is in good agreement with our results, but the CCSD(T)/cc-pVTZ//M06-2X/6-31+G(*d*,*p*) transition state barrier of 2.25 kcal mol^–1^ is 1.78 kcal mol^–1^ higher than our transition state barrier. The G3(MP2)//M06-2X/6-31+G(*d*,*p*) pathway predicts a submerged transition state barrier of −1.43 kcal mol^–1^. The transition state geometries are quite similar and the largest difference is the CHC angle again. Rate constants were reported by Dash and Rajakumar and will be discussed in the Kinetics section.

### 3.2 Kinetics

The rate constants computed in this study using the rigid-rotor harmonic oscillator approximation can be found in Table 7. The Theoretical Methods section contains the methods used for obtaining these rate constants. The abstractions from *cis*-HONO and CH_3_OH (both reactions R1 and R2) have submerged barriers, and as such the rate constants for these reaction will likely be large at all temperatures. Because of this, we have only examined the rate constants for the abstractions from HNCO, *trans*-HONO, and C_2_H_4_.

**Table 7:** Rate Constants for C_2_H + HX → C_2_H_2_ + X Abstractions in cm^3^ molecule^–1^ s^–1^

*T* (K)	HNCO	*trans*-HONO	C_2_H_4_
50	3.93 × 10^–16^	6.20 × 10^–18^	2.45 × 10^–14^
100	1.06 × 10^–15^	8.39 × 10^–18^	1.07 × 10^–13^
150	3.22 × 10^–15^	1.66 × 10^–17^	2.67 × 10^–13^
175	5.33 × 10^–15^	2.46 × 10^–17^	3.77 × 10^–13^
200	8.41 × 10^–15^	3.66 × 10^–17^	5.07 × 10^–13^
225	1.27 × 10^–14^	5.44 × 10^–17^	6.57 × 10^–13^
250	1.84 × 10^–14^	8.02 × 10^–17^	8.29 × 10^–13^
275	2.57 × 10^–14^	1.17 × 10^–16^	1.02 × 10^–12^
295	3.29 × 10^–14^	1.56 × 10^–16^	1.19 × 10^–12^
298	3.40 × 10^–14^	1.62 × 10^–16^	1.22 × 10^–12^
300	3.48 × 10^–14^	1.67 × 10^–16^	1.24 × 10^–12^
325	4.61 × 10^–14^	2.34 × 10^–16^	1.48 × 10^–12^
350	5.95 × 10^–14^	3.22 × 10^–16^	1.74 × 10^–12^
375	7.55 × 10^–14^	4.35 × 10^–16^	2.03 × 10^–12^
400	9.40 × 10^–14^	5.76 × 10^–16^	2.35 × 10^–12^
500	1.99 × 10^–13^	1.52 × 10^–15^	3.90 × 10^–12^
1000	1.88 × 10^–12^	2.58 × 10^–14^	2.10 × 10^–11^
1500	6.78 × 10^–12^	1.17 × 10^–13^	6.03 × 10^–11^
2000	1.66 × 10^–11^	3.23 × 10^–13^	1.29 × 10^–10^
3000	5.68 × 10^–11^	1.25 × 10^–12^	3.82 × 10^–10^
4000	1.32 × 10^–10^	3.10 × 10^–12^	8.19 × 10^–10^
5000	2.51 × 10^–10^	6.10 × 10^–12^	1.47 × 10^–9^

Quantitatively accurate kinetic models require highly accurate barrier heights of reaction. This makes the rate constants highly dependent on the calculated barrier heights. Additionally, the reaction barriers are low, therefore, variational effects are likely to be important which could be beneficial to explore in a future study.

#### 3.2.1 C_2_H + C_2_H_4_

**Figure 10:**
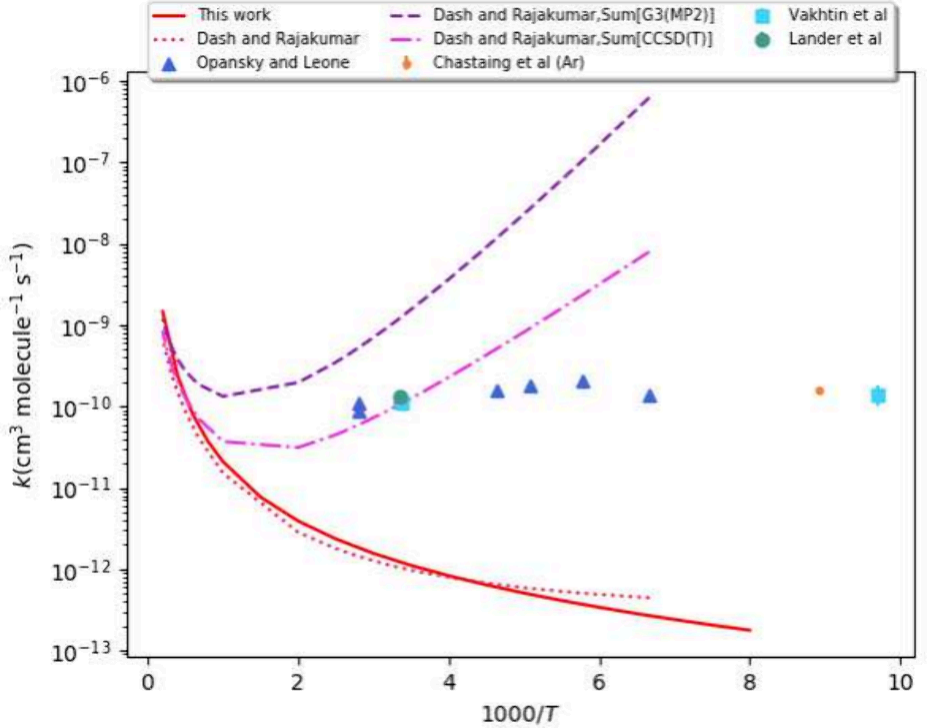
Experimental and theoretical rate constants for the C_2_H + C_2_H_4_ → C_2_H_2_ + C_2_H_3_ reaction. Theoretical rate constants are illustrated as solid curves and experimental rate constants are given as points.^9,18,20,72,73^

The computed rate constants from this study for the C_2_H + C_2_H_4_ hydrogen abstraction reaction are plotted in Figure 10 (solid red line). Additionally, the canonical variational transition state theory (CVT) theoretical rate constants of Dash and Rajakumar, as well as various experimental rate constants have been included. The rate constants of Dash and Rajakumar were obtained from the sum of the individual rate coefficients associated with abstraction (C_2_H + C_2_H_4_ → C_2_H_2_ + C_2_H_3_) and addition (C_2_H + C_2_H_4_ → C_2_H_4_CCH) channels. The sum of the individual rate coefficients at the G3(MP2)//M06-2X/6-31+G(*d*,*p*) and CCSD(T)/cc-pVTZ//M06-2X/6-31+G(*d*,*p*) levels of theory are given by the dashed purple line and dashed pink line, respectively. The rate constants for only the abstraction channel by Dash and Rajakumar at the CCSD(T)/cc-pVTZ//M06-2X/6-31+G(*d*,*p*) level of theory are given by the red dotted line. Our computed rate constants for the abstraction channel are in good agreement with theirs at the CCSD(T)/cc-pVTZ//M06-2X/6-31+G(*d*,*p*) level of theory. Dash and Rajakumar observed a strong negative temperature dependence for their computed rate constants, and the hydrogen abstraction contribution to the total rate constant is negligible below 250 K. Above 1000 K, the abstraction and addition reactions are in competition with each other. However, this competition is outside of the scope of the present study but it could be beneficial to explore the effects in the future. A negative temperature dependence was also reported in earlier experimental studies, but not to the same degree as that of Dash and Rajakumar. The experiments were performed using supersonic expansion methods. Opansky and Leone used transient infrared laser absorption spectroscopy,^9^ Chastaing et. al used CRESU (laval nozzle expansion),^72^ and Vakhtin et al. used pulsed nozzle expansion methods.^73^

## 4 Conclusion

The energetics of ethynyl radical hydrogen abstraction reactions involving HNCO, *trans*-HONO, *cis*-HONO, C_2_H_4_, and CH_3_OH have been determined using highly accurate *ab initio* methods. Subchemical accuracy was achieved through various additive energy corrections, and shows excellent agreement with the available Active Thermochemical Table values. Additionally, accurate transition state barriers have been determined for the reactions involving HNCO, *trans*-HONO, C_2_H_4_, and CH_3_OH (reaction R1) in this study. The reaction pathways involving *cis*-HONO and CH_3_OH (reaction R2) have been determined at the CCSD(T)-F12a/cc-pVTZ-F12//MP2/aug-cc-pVTZ level of theroy. The reactions with CH_3_OH (reactions R1 and R2) and *cis*-HONO have submerged barriers below the relative enthalpies of the reactants. Abstractions of *trans*-HONO, HNCO, and C_2_H_4_ have barriers between 0.5 and 5.0 kcal mol^–1^. The reactions appear to follow the Evans–Polanyi principle and a strong correlation between the barrier height and reaction enthalpy was seen. One exception to the Evans–Polanyi principle was found with the C_2_H + *trans*-HONO reaction, which is believed to be due to the interaction between the nitrogen in *trans*-HONO and the terminal carbon of the ethynyl radical. This interaction raises the barrier height of the transition state to almost 5 kcal mol^–1^.

Reliable kinetics were obtained for a subset of the above reactions implementing an Eckart tunnelling model. The computed rate constants for the C_2_H + C_2_H_4_ → C_2_H_2_ + C_2_H_3_ reaction are in good agreement with those computed by Dash and Rajakumar. However, there appears to be a potential competition between the abstraction and addition channels of this reaction which could explain the disagreement between the computed rate constants and experiment. The kinetics of the reactions with *trans*-HONO and HNCO have not yet been explored, so the results presented in this study may aid in future experimental studies.

## Supporting Information Available

Cartesian coordinates, harmonic vibrational frequencies, rotational constants, and wave function diagnostics (PDF)
