# Supplementary material for: Ethynyl Radical Hydrogen Abstraction Energetics and Kinetics Utilizing High-Level Theory
Source: ACS Earth Space Chem. 2024 Jul 3;8(7):1349–58. doi: 10.1021/acsearthspacechem.4c00040 (PMC11261607; doi:10.1021/acsearthspacechem.4c00040)
Supplement: Supplementary file 1 — sp4c00040_si_002.pdf [file sp4c00040_si_002.pdf]

Ethynyl Radical Hydrogen Abstraction Energetics and Kinetics Utilizing High-Level Theory Supporting Information:

Laura N. Olive
Alexandra D. Heide
Justin M. Turney
Henry F. Schaefer III*

Center for Computational Quantum Chemistry
University of Georgia
Athens, GA, USA 30602

E-mail: ccq@uga.edu

Contents

1 Reactants **S3**

1.1 C_2H ($^2\Sigma^+$) S3
1.2 HNCO S4
1.3 *trans*-HONO S5
1.4 *cis*-HONO S6
1.5 CH_3OH S7
1.6 C_2H_4 . . . . . S8

2 Products **S9**

2.1 C_2H_2 . . . . . S9
2.2 NCO S10
2.3 NO_2 . . . . . S11
2.4 CH_3O S12
2.5 CH_2OH S13
2.6 C_2H_3 . . . . . S14

3 Transition States **S15**

3.1 C_2H + HNCO S15
3.2 C_2H + *cis*-HONO S16
3.3 C_2H + *trans*-HONO S17
3.4 C_2H + CH_3OH (R1) S18
3.5 C_2H + CH_3OH (R2) S19
3.6 C_2H + C_2H_4 . . . . . S20

4 Pre-reactive complex **S21**

4.1 C_2H + CH_3OH (R1) S21
4.2 C_2H + CH_3OH (R2) S22
4.3 C_2H + *cis*-HONO S23

1 Reactants

1.1 C_2H ($^2\Sigma^+$)

Level of Theory:

Reference:	ROHF
Geometry:	CCSD(T)-F12a/cc-pVTZ-F12
Frequencies:	CCSD(T)-F12a/cc-pVTZ-F12
Program:	Molpro 2010.1

Wavefunction Diagnostics:

T_1: 0.014

D_1: 0.033

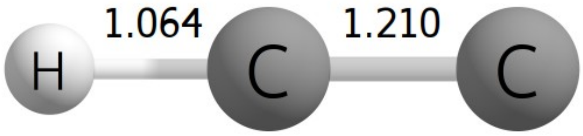

Figure S1: C_2H

Cartesian Coordinates (Å):

```
C     0.000000000000      0.000000000000     -0.537842251700
C     0.000000000000      0.000000000000      0.672292694400
H     0.000000000000      0.000000000000     -1.602163091700
```

Rotational Constants (GHz): 43.9823985, 43.9823985

Harmonic Vibrational Frequencies (cm^{-1}):

σ^+: 3445 2021

π: 375

1.2 HNCO

Level of Theory:

Reference:	ROHF
Geometry:	CCSD(T)-F12a/cc-pVTZ-F12
Frequencies:	CCSD(T)-F12a/cc-pVTZ-F12
Program:	MOLPRO 2010.1

Wavefunction Diagnostics:

T_1: 0.015

D_1: 0.051

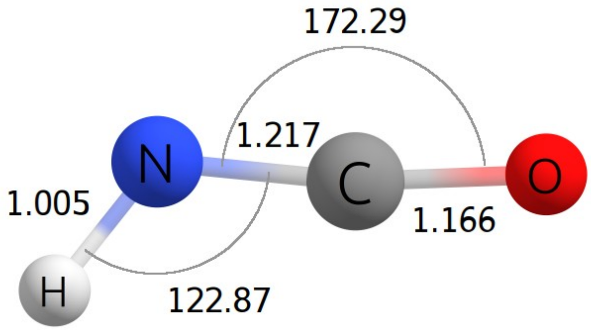

Figure S2: HNCO

Cartesian Coordinates (Å):

```
N     0.000000000000     -0.080565763200     -1.207331025500
C     0.000000000000      0.038992777400      0.004189838700
H     0.000000000000      0.705936148900     -1.833005510400
O     0.000000000000     -0.003214061500      1.169291279400
```

Rotational Constants (GHz): 10.9175915, 11.0637799, 826.2616391

Harmonic Vibrational Frequencies (cm^{-1}):

a':	570	821	1311	2303	3686
a":	633				

1.3 *trans*-HONO

Level of Theory:

Reference:	ROHF
Geometry:	CCSD(T)-F12a/cc-pVTZ-F12
Frequencies:	CCSD(T)-F12a/cc-pVTZ-F12
Program:	MOLPRO 2010.1

Wavefunction Diagnostics:

T_1: 0.018

D_1: 0.060

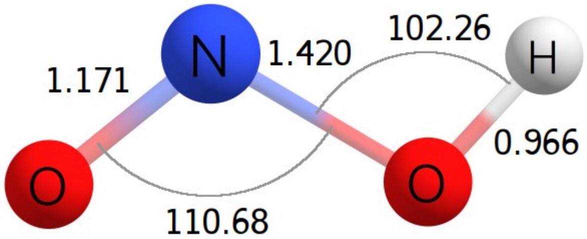

Figure S3: *trans*-HONO

Cartesian Coordinates (Å):

```
N      0.000000000000     -0.495195417300     -0.146683289600
O      0.000000000000      0.224957555200      1.077562688700
O      0.000000000000      0.239107640800     -1.058334582400
H      0.000000000000     -0.484861246200      1.733149461900
```

Rotational Constants (GHz): 11.1752351, 12.6893335, 93.6572421

Harmonic Vibrational Frequencies (cm^{-1}):

a':	634	834	1317	1731	3777
a":	573				

1.4 *cis*-HONO

Level of Theory:

Geometry:	MP2/aug-cc-pVTZ
Frequencies:	MP2/aug-cc-pVTZ
Program:	Psi4

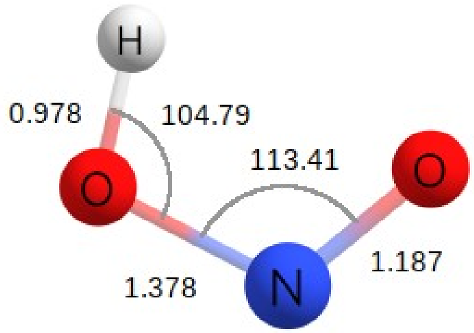

Figure S4: *cis*-HONO

Cartesian Coordinates (Å):

N	-0.140692248224	0.500940475242	0.000000000000
O	-0.891950927124	-0.653983241826	0.000000000000
O	1.029955331231	0.302312246545	0.000000000000
H	-0.235402663668	-1.378968529740	0.000000000000

Rotational Constants (GHz): 84.47242506, 13.378268, 11.54917756

Harmonic Vibrational Frequencies (cm^{-1}):

a':	645	896	1329	1628	3608
a":	701				

1.5 CH_3OH

Level of Theory:

Reference:	ROHF
Geometry:	CCSD(T)-F12a/cc-pVTZ-F12
Frequencies:	CCSD(T)-F12a/cc-pVTZ-F12
Program:	MOLPRO 2010.1

Wavefunction Diagnostics:

T_1: 0.009

D_1: 0.021

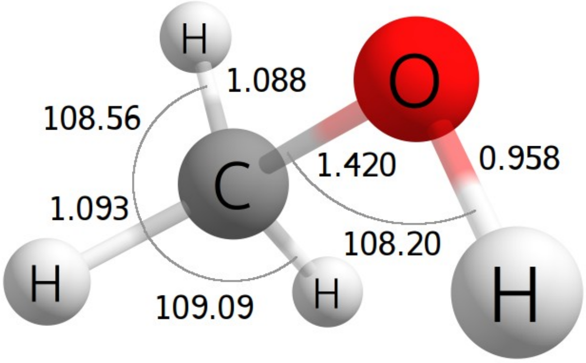

Figure S5: CH_3OH

Cartesian Coordinates (Å):

```
C      0.000000000200     -0.013122552500     -0.728062632300
H      0.000000006000      1.009824433100     -1.097174950600
H     -0.890375835400     -0.520290717500     -1.108648177800
H      0.890375826000     -0.520290734500     -1.108648178400
O      0.000000000300      0.063998070000      0.689965825900
H     -0.000000004700     -0.828734362900      1.038265422500
```

Rotational Constants (GHz): 23.9580654, 24.8233147, 128.5452844

Harmonic Vibrational Frequencies (cm^{-1}):

a: 293 1061 1089 1181 1382 1484 1511 1521 3016 3076 3137 3864

1.6 C_2H_4

Level of Theory:

Reference:	ROHF
Geometry:	CCSD(T)-F12a/cc-pVTZ-F12
Frequencies:	CCSD(T)-F12a/cc-pVTZ-F12
Program:	Molpro 2010.1

Wavefunction Diagnostics:

T_1: 0.010

D_1: 0.035

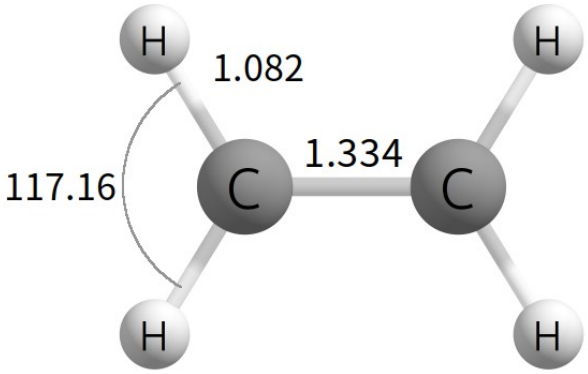

Figure S6: C_2H_4

Cartesian Coordinates (Å):

```
H    -1.446173188000     -0.526428925500      0.000000000000
C    -0.364314854100     -0.558500161700      0.000000000000
C     0.364313435200      0.558501077100      0.000000000000
H    -0.101036054600      1.535684425800      0.000000000000
H     1.446166868900      0.526402477300      0.000000000000
H     0.101062021900     -1.535669798100      0.000000000000
```

Rotational Constants (GHz): 24.9943280, 30.1169507, 146.9467880

Harmonic Vibrational Frequencies (cm^{-1}):

a':	825	1249	1368	1478	1671	3141	3155	3222	3248
a"	950	964	1050						

2 Products

2.1 C_2H_2

Level of Theory:

Reference:	ROHF
Geometry:	CCSD(T)-F12a/cc-pVTZ-F12
Frequencies:	CCSD(T)-F12a/cc-pVTZ-F12
Program:	Molpro 2010.1

Wavefunction Diagnostics:

T_1: 0.012
D_1: 0.031

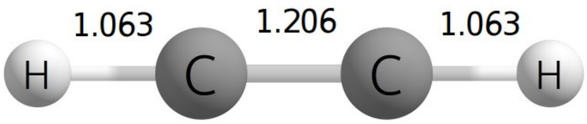

Figure S7: C_2H_2

Cartesian Coordinates (Å):

```
C     0.000000000000     0.000000000000    -0.602785143000
C     0.000000000000     0.000000000000     0.602785143100
H     0.000000000000     0.000000000000     1.666005547000
H     0.000000000000     0.000000000000    -1.666005547300
```

Rotational Constants (GHz): 35.2829171, 35.2829171

Harmonic Vibrational Frequencies (cm^{-1}):

σ_g^+:	3502	2007
σ_u^+:	3410	
π_g:	618	
π_u:	749	

2.2 NCO

Level of Theory:

Reference:	ROHF
Geometry:	CCSD(T)-F12a/cc-pVTZ-F12
Frequencies:	CCSD(T)-F12a/cc-pVTZ-F12
Program:	Molpro 2010.1

Wavefunction Diagnostics:

T_1: 0.028

D_1: 0.109

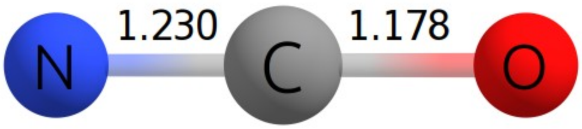

Figure S8: NCO

Cartesian Coordinates (Å):

```
O      0.000000000000      0.000000000000     -1.139022616400
C      0.000000000000      0.000000000000      0.038505406300
N      0.000000000000      0.000000000000      1.268049577200
```

Rotational Constants (GHz): 11.6723629, 11.6723629

Harmonic Vibrational Frequencies (cm^{-1}):

a_1:	1977	1269
b_2:	581	
b_1:	502	

2.3 NO_2

Level of Theory:

Reference:	ROHF
Geometry:	CCSD(T)-F12a/cc-pVTZ-F12
Frequencies:	CCSD(T)-F12a/cc-pVTZ-F12
Program:	MOLPRO 2010.1

Wavefunction Diagnostics:

T_1: 0.022

D_1: 0.065

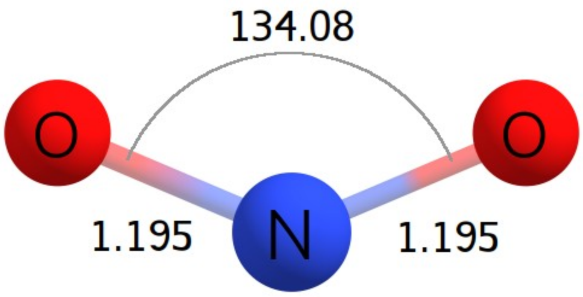

Figure S9: NO_2

Cartesian Coordinates (Å):

```
O     0.000000000000    -1.100370062100     0.141917351000
N     0.000000000000     0.000000000000    -0.324215192000
O     0.000000000000     1.100370062100     0.141917351000
```

Rotational Constants (GHz): 12.3681279, 238.7473577, 13.0438550

Harmonic Vibrational Frequencies (cm^{-1}):

a_1: 762 1359

b_2: 1682

2.4 CH_3O

Level of Theory:

Reference:	ROHF
Geometry:	CCSD(T)-F12a/cc-pVTZ-F12
Frequencies:	CCSD(T)-F12a/cc-pVTZ-F12
Program:	MOLPRO 2010.1

Wavefunction Diagnostics:

T_1: 0.018

D_1: 0.064

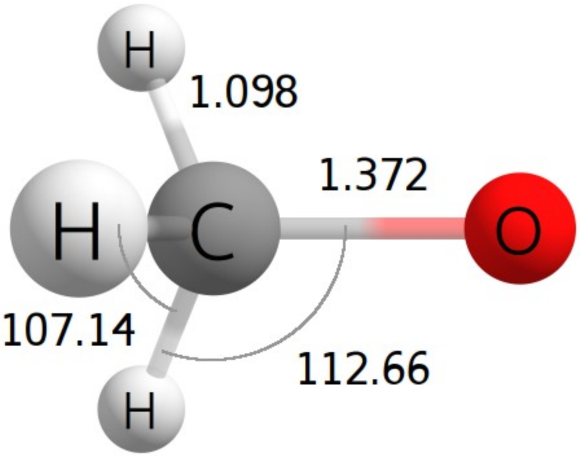

Figure S10: CH_3O

Cartesian Coordinates (Å):

```
C    -0.006656777600      0.000000000000     -0.670480265700
O    -0.003790569800      0.000000000000      0.701347309300
H    -0.456511994100      0.907823768900     -1.092463965900
H     1.052517805600      0.000000000000     -0.958113985200
H    -0.456511994100     -0.907823768900     -1.092463965900
```

Rotational Constants (GHz): 27.6738970, 27.8629479, 157.9880589

Harmonic Vibrational Frequencies (cm^{-1}):

a':	963	1111	1387	1522	2956	3029
a":	739	1388	3040			

2.5 CH_2OH

Level of Theory:

Geometry:	MP2/aug-cc-pVTZ
Frequencies:	MP2/aug-cc-pVTZ
Program:	Psi4

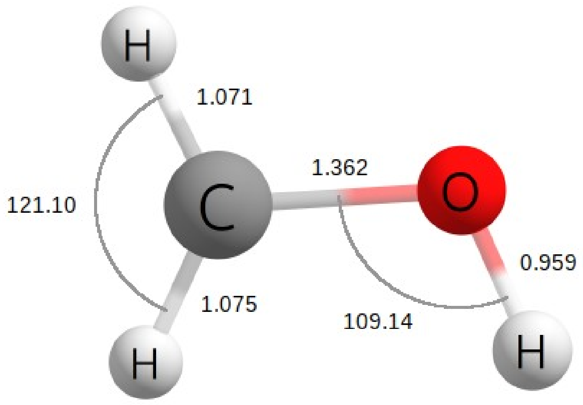

Figure S11: CH_2OH

Cartesian Coordinates (Å):

C	0.043479616321	0.016898362841	-0.725175178004
O	-0.015235115208	-0.065950524015	0.632848136852
H	0.065712859878	0.814222329740	1.005489458859
H	-0.207738783258	0.949391499703	-1.197328907065
H	-0.133886085146	-0.918137065722	-1.217383119826

Rotational Constants (GHz): 195.03213383, 30.14272781, 26.35865559

Harmonic Vibrational Frequencies (cm^{-1}):

a: 430 619 1073 1222 1374 1520 3219 3340 3880

2.6 C_2H_3

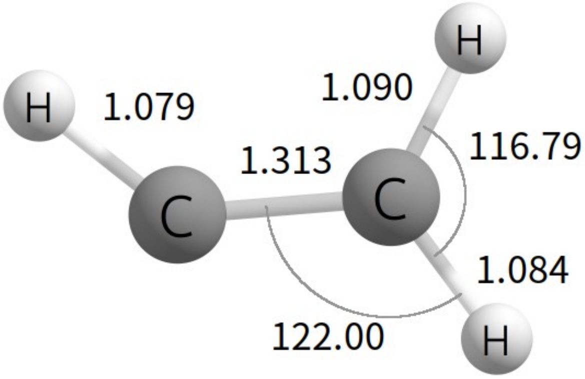

Figure S12: C_2H_3

Level of Theory:

Reference:	ROHF
Geometry:	CCSD(T)-F12a/cc-pVTZ-F12
Frequencies:	CCSD(T)-F12a/cc-pVTZ-F12
Program:	MOLPRO 2010.1

Wavefunction Diagnostics:

T_1: 0.015

D_1: 0.037

Cartesian Coordinates (Å):

```
C    -0.365263420700     -0.590682476300      0.000000000000
C     0.279716790300      0.552681348200      0.000000000000
H    -0.238899499900      1.504962138800      0.000000000000
H     1.369156015000      0.586506548500      0.000000000000
H    -0.111669804600     -1.638995384000      0.000000000000
```

Rotational Constants (GHz): 233.6654524, 32.5517429, 28.5714742

Harmonic Vibrational Frequencies (cm^{-1}):

a':	722	1069	1392	1618	3071	3177	3249
a":	809	911					

3 Transition States

3.1 C_2H + HNCO

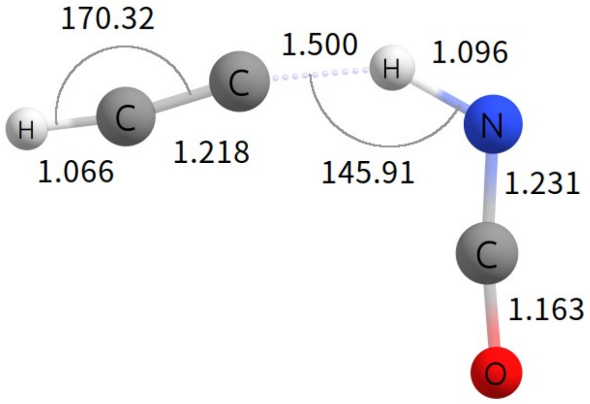

Figure S13: C_2H + HNCO

Level of Theory:

Reference:	ROHF
Geometry:	CCSD(T)-F12a/cc-pVTZ-F12
Frequencies:	CCSD(T)-F12a/cc-pVTZ-F12
Program:	MOLPRO 2010.1

Wavefunction Diagnostics:

T_1: 0.026

D_1: 0.095

Cartesian Coordinates (Å):

```
O    -0.872375158600     0.000000000000     1.902564774200
C     0.077611285500     0.000000000000     1.232060696000
N     1.163117470300     0.000000000000     0.650863886900
H     1.155581215600     0.000000000000    -0.445099605700
C     0.306348662000     0.000000000000    -1.681259489700
C    -0.573781469900     0.000000000000    -2.523215161800
H    -1.209167791600     0.000000000000    -3.379221320100
```

Rotational Constants (GHz): 2.4748091, 2.0771172, 12.9257552

Harmonic Vibrational Frequencies (cm^{-1}):

a':	158	416	584	742	1273	1713	1993	2237	3425	947i
a":	43	92	408	556	617					

3.2 C_2H + *cis*-HONO

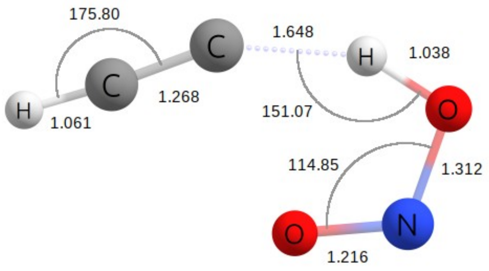

Figure S14: C_2H + *cis*-HONO

Level of Theory:

Geometry:	MP2/aug-cc-pVTZ
Frequencies:	MP2/aug-cc-pVTZ
Program:	Psi4

Cartesian Coordinates (Å):

```
O     -0.938193288035     0.303992424446     0.001060047149
N     -0.350085191439     1.368614535539     0.000643795714
O      0.958599546775     1.275633633673    -0.000373453603
H      1.145212001793     0.254666140171    -0.000526147679
H     -0.909478308571    -3.061159596861    -0.000277234091
C     -0.244678109395    -2.234539013350    -0.000269638243
C      0.620331726235    -1.307265852142    -0.000257369246
```

Rotational Constants (GHz): 13.53341071, 3.50240906, 2.78234586

Harmonic Vibrational Frequencies (cm^{-1}):

a: $190i$ 93 177 239 259 603 658 764 1093 1113 1399 1583 1793 2456 3449

3.3 C_2H + *trans*-HONO

Level of Theory:

Reference:	ROHF
Geometry:	CCSD(T)-F12a/cc-pVTZ-F12
Frequencies:	CCSD(T)-F12a/cc-pVTZ-F12
Program:	MOLPRO 2010.1

Wavefunction Diagnostics:

T_1: 0.063

D_1: 0.307

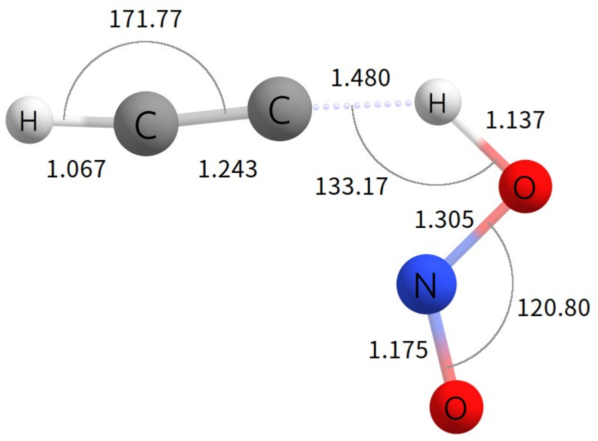

Figure S15: C_2H + *trans*-HONO

Cartesian Coordinates (Å):

```
H     0.000000000000     -1.024399249200     -3.216384019500
C     0.000000000000     -0.437919922900     -2.324510242600
C     0.000000000000      0.386338738900     -1.394729710400
H     0.000000000000      1.293024481800     -0.225032035300
O     0.000000000000      1.114292044200      0.897764154500
N     0.000000000000     -0.167873490100      0.656353055700
O     0.000000000000     -0.945527146900      1.536526608100
```

Rotational Constants (GHz): 2.5673533, 3.2511744, 12.2062832

Harmonic Vibrational Frequencies (cm^{-1}):

a':	101	245	504	660	805	1162	1620	1814	2062	3404	1703i
a":	126	288	629	1133							

3.4 $C_2H + CH_3OH$ (R1)

Level of Theory:

Reference:	ROHF
Geometry:	CCSD(T)-F12a/cc-pVTZ-F12
Frequencies:	CCSD(T)-F12a/cc-pVTZ-F12
Program:	Molpro 2010.1

Wavefunction Diagnostics:

T_1: 0.033
D_1: 0.147

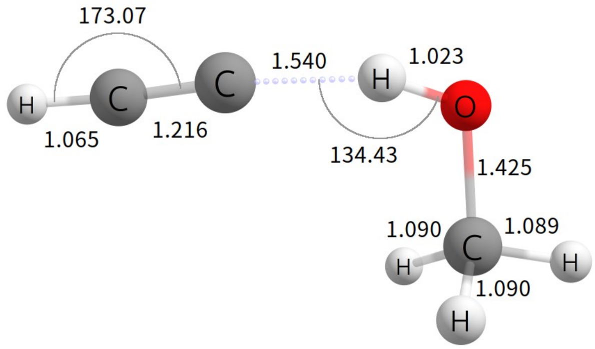

Figure S16: $C_2H + CH_3OH$

Cartesian Coordinates (Å):

```
C    -0.136796049012      0.135383475507      1.185529601444
C     0.459300224707     -0.074977375807      2.224725919857
H     0.927382642575     -0.150819905546      3.178713334397
H    -0.758256372822      0.405906302831     -0.197791738751
O    -0.773529985043     -0.099386587801     -1.087423610640
C     0.492157269397      0.035565343791     -1.728798944281
H     0.310791175159     -0.191645382595     -2.778505392259
H     1.213451564241     -0.677865316414     -1.330184724393
H     0.883078928035      1.049046036957     -1.634845055467
```

Rotational Constants (GHz): 23.9580654, 24.8233147, 128.5452844

Harmonic Vibrational Frequencies (cm^{-1}):

a:	68	85	125	212	283	569	585	1027	1070	1171	1294
	1461	1484	1515	1940	2323	3037	3119	3133	3433	794i	

3.5 $C_2H + CH_3OH$ (R2)

Level of Theory:

Geometry:	MP2/aug-cc-pVTZ
Frequencies:	MP2/aug-cc-pVTZ
Program:	Psi4

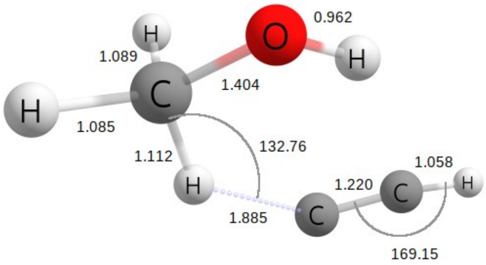

Figure S17: $C_2H + CH_3OH$

Cartesian Coordinates (Å):

C	-0.307852120223	1.507211038556	0.209602522559
C	-1.386363987615	1.451012374396	-0.357775919536
H	1.004208781999	0.228904772617	0.652731219622
C	1.307659422490	-0.734101576948	0.187067594326
O	0.191365197660	-1.547927085382	-0.064354146998
H	1.868927476914	-0.498746475747	-0.715928024675
H	1.945536320760	-1.247557255465	0.898945974015
H	-0.479195119249	-0.991254603452	-0.471119667586
H	-2.342624979635	1.591259668514	-0.787595823113

Rotational Constants (GHz): 17.50558078, 3.59195079, 3.06225028

Harmonic Vibrational Frequencies (cm^{-1}):

a:	303*i*	63	89	151	173	340	474	498	1063	1091	1182	1375	1454
	1480	1523	1874	2676	3092	3173	3479	3828					

3.6 $C_2H + C_2H_4$

Level of Theory:

Reference:	ROHF
Geometry:	CCSD(T)-F12a/cc-pVTZ-F12
Frequencies:	CCSD(T)-F12a/cc-pVTZ-F12
Program:	MOLPRO 2010.1

Wavefunction Diagnostics:

T_1: 0.019

D_1: 0.064

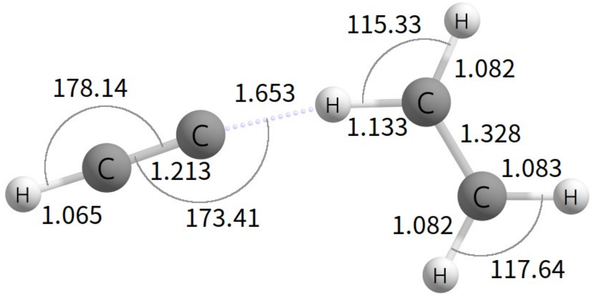

Figure S18: $C_2H + C_2H_4$

Cartesian Coordinates (Å):

```
H    -3.475782783100      0.598247085600      0.000000000000
C    -2.467378185900      0.256570090800      0.000000000000
C    -1.331987159300     -0.169697077100      0.000000000000
H     0.272054861600     -0.569131967900      0.000000000000
C     1.403818902500     -0.622064219500      0.000000000000
C     2.107514997600      0.504456965800      0.000000000000
H     1.621847777000      1.471654917000      0.000000000000
H     3.190767101500      0.486014855800      0.000000000000
H     1.820651882200     -1.620892312500      0.000000000000
```

Rotational Constants (GHz): 34.83446821, 2.528453954, 2.357346368

Harmonic Vibrational Frequencies (cm^{-1}):

a':	269i	33	116	548	791	1190	1331	1431	1649	1936	2107	3141	3198	3235	3440
a":	44	167	543	907	942	975									

4 Pre-reactive complex

4.1 $C_2H + CH_3OH$ (R1)

Level of Theory:

Geometry:	CCSD(T)-F12a/cc-pVTZ-F12
Frequencies:	CCSD(T)-F12a/cc-pVTZ-F12
Program:	MOLPRO 2010.1

Wavefunction Diagnostics:

T_1: 0.023

D_1: 0.091

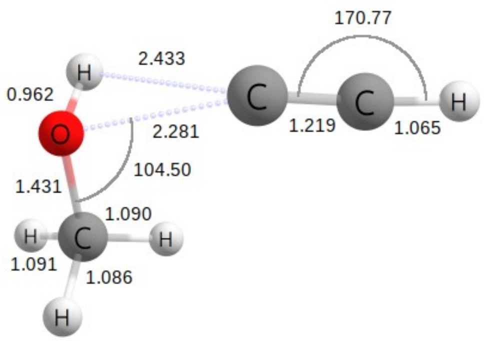

Figure S19: $C_2H + CH_3OH$ (R1)

Cartesian Coordinates (Å):

```
C     0.970275482838      0.772730881527     -0.305920740993
C     0.930053249848      1.874295133478      0.215459614495
H     0.298958920666     -1.273317841604      0.827418946689
O    -0.070673827321     -1.241440200420     -0.059996239182
C    -1.460131327862     -0.916483461871      0.042118111452
H    -2.004002569625     -1.721353159484      0.539425381860
H    -1.826363077430     -0.809212313977     -0.975052281726
H    -1.606571848696      0.022631682243      0.576534062920
H     1.018268415437      2.878534794926      0.559469741851
```

Harmonic Vibrational Frequencies (cm^{-1}):

a: 20 81 119 180 218 443 551 558 1038 1076 1176 1367 1473 1507 1513
1941 3037 3112 3157 3431 3819

4.2 $C_2H + CH_3OH$ (R2)

Level of Theory:

Geometry:	MP2/aug-cc-pVTZ
Frequencies:	MP2/aug-cc-pVTZ
Program:	Psi4

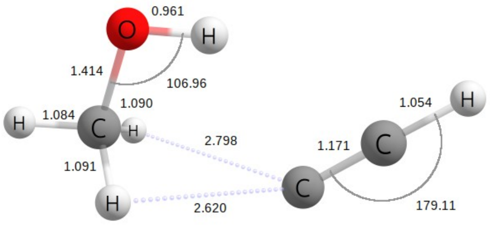

Figure S20: $C_2H + CH_3OH$

Cartesian Coordinates (Å):

```
C     -0.355155442445    1.529473387116   -0.001292256334
C     -1.465173496488    1.548887531847   -0.375119579169
H      1.227932841668   -0.191167710510    1.180128315340
C      1.373025737357   -0.853769649700    0.325336468332
O      0.200524409641   -1.581480595426    0.016330246238
H      1.724758524212   -0.267754769410   -0.524259205395
H      2.140142695206   -1.574755760819    0.585103806704
H     -0.485135438269   -0.937617368450   -0.181681439342
H     -2.464203930311    1.582713176663   -0.710318688857
```

Rotational Constants (GHz): 18.55029149, 3.20695739, 2.78360581

Harmonic Vibrational Frequencies (cm^{-1}):

a: $32i$ 69 111 134 140 428 811 812 1075 1104 1195 1400 1493 1536 1544 2541 3048 3095 3173 3588 3837

4.3 C_2H + *cis*-HONO

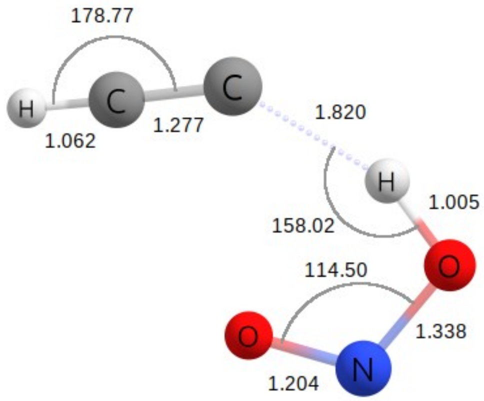

Figure S21: C_2H + *cis*-HONO

Level of Theory:

Geometry:	MP2/aug-cc-pVTZ
Frequencies:	MP2/aug-cc-pVTZ
Program:	Psi4

Cartesian Coordinates (Å):

O	-0.995852844159	0.426239342892	0.001046306245
N	-0.373711820944	1.456752006277	0.000347180268
O	0.955782449855	1.302547919964	-0.000028808549
H	1.125753588560	0.311822591363	-0.000015238219
H	-1.002736021723	-3.026363202312	-0.000241295739
C	-0.220143716887	-2.309144291749	-0.000705799030
C	0.739635782330	-1.466722473635	-0.001280683891

Rotational Constants (GHz): 12.08098927, 3179.79488, 2517.24096

Harmonic Vibrational Frequencies (cm^{-1}):

a: 83 95 156 203 217 591 635 727 969 1036 1433 1596 1765 3045 3442
